# The Role of HDAC6 in TDP-43-Induced Neurotoxicity and UPS Impairment

**DOI:** 10.3389/fcell.2020.581942

**Published:** 2020-11-17

**Authors:** Shinrye Lee, Younghwi Kwon, Seyeon Kim, Myungjin Jo, Yu-Mi Jeon, Mookyung Cheon, Seongsoo Lee, Sang Ryong Kim, Kiyoung Kim, Hyung-Jun Kim

**Affiliations:** ^1^Dementia Research Group, Korea Brain Research Institute, Daegu, South Korea; ^2^Department of Brain and Cognitive Sciences, DGIST, Daegu, South Korea; ^3^Gwangju Center, Korea Basic Science Institute (KBSI), Gwangju, South Korea; ^4^School of Life Sciences, BK21 Plus KNU Creative BioResearch Group, Institute of Life Science and Biotechnology, Kyungpook National University, Daegu, South Korea; ^5^Brain Science and Engineering Institute, Kyungpook National University, Daegu, South Korea; ^6^Department of Medical Biotechnology, Soonchunhyang University, Asan, South Korea

**Keywords:** tar DNA-binding protein 43, histone deacetylase 6, ubiquitin-proteasome system, amyotrophic lateral sclerosis, autophagy-lysosome pathway

## Abstract

Transactive response DNA-binding protein 43 (TDP-43)-induced neurotoxicity is currently well recognized as a contributor to the pathology of amyotrophic lateral sclerosis (ALS), and the deposition of TDP-43 has been linked to other neurodegenerative diseases, such as frontotemporal lobar degeneration (FTLD) and Alzheimer’s disease (AD). Recent studies also suggest that TDP-43-induced neurotoxicity is associated with ubiquitin-proteasome system (UPS) impairment. Histone deacetylase 6 (HDAC6) is a well-known cytosolic deacetylase enzyme that suppresses the toxicity of UPS impairment. However, the role of HDAC6 in TDP-43-induced neurodegeneration is largely unknown. In this study, we found that HDAC6 overexpression decreased the levels of insoluble and cytosolic TDP-43 protein in TDP-43-overexpressing N2a cells. In addition, TDP-43 overexpression upregulated HDAC6 protein and mRNA levels, and knockdown of *Hdac6* elevated the total protein level of TDP-43. We further found that HDAC6 modulates TDP-43-induced UPS impairment via the autophagy-lysosome pathway (ALP). We also showed that TDP-43 promoted a short lifespan in flies and that the accumulation of ubiquitin aggregates and climbing defects were significantly rescued by overexpression of HDAC6 in flies. Taken together, these findings suggest that HDAC6 overexpression can mitigate neuronal toxicity caused by TDP-43-induced UPS impairment, which may represent a novel therapeutic approach for ALS.

## Introduction

Transactive response DNA-binding protein 43 (TDP-43) is an evolutionarily conserved member of the heterogeneous nuclear ribonucleoprotein (hnRNP) family, and it is encoded by the *TARDBP* gene (Ou et al., 1995). Previous studies have revealed that TDP-43 regulates RNA metabolic processes, such as transcription, splicing, translation, and regulating stability of mRNA (Strong et al., 2007; Buratti and Baralle, 2008). TDP-43 is predominantly localized in the nucleus, but it can shuttle between the nucleus and the cytoplasm (Barmada et al., 2010). The accumulation of misfolded or aggregated protein in affected neurons is a major pathological feature in most neurodegenerative diseases. It is already known that cytoplasmic accumulation of TDP-43 aggregates is one of the major characteristics of TDP-43 proteinopathy (Kim et al., 2014; Scotter et al., 2015; Shenouda et al., 2018), and this is a common pathological feature associated with many neurodegenerative diseases, such as Alzheimer’s disease (AD), frontotemporal lobar degeneration (FTLD), and amyotrophic lateral sclerosis (ALS) (Neumann et al., 2006; Mackenzie et al., 2011; Tremblay et al., 2011). Many neuropathological mechanisms, such as oxidative stress, mitochondrial dysfunction, neuroinflammation, and ER stress, are linked to TDP-43 proteinopathy (Duan et al., 2010; Kim et al., 2014; Zhao et al., 2015; Wang et al., 2019). In particular, emerging clinical and experimental evidence suggests that dysfunctions in protein quality control, including problems with the ubiquitin-proteasome system (UPS) impairment, are the core pathological mechanism of TDP-43-mediated neurodegeneration (Scotter et al., 2014; Lee et al., 2019).

Histone acetyltransferases (HATs) and histone deacetylases (HDACs) are two classes of enzymes whose opposing activities regulate the dynamic levels of acetylation of specific lysine residues in histones and many other proteins (Yang and Seto, 2007). Histone deacetylase 6 (HDAC6) is a member of the class II HDAC family, and it contains a ubiquitin binding domain. Unlike other HDACs, HDAC6 targets non-histone substrates, such as α-tubulin, heat shock protein 90 (HSP90), and cortactin, to modulate widespread biological processes (Zhang et al., 2003, 2007; Kovacs et al., 2005). HDAC6 contains a ubiquitin-binding domain that binds to ubiquitinated proteins for degradation. In a previous study, HDAC6 rescued proteasome impairment in *Drosophila* (Pandey et al., 2007). Additionally, HDAC6 controls the autophagy-lysosome pathway (ALP). HDAC6 is not required for autophagy activation; rather, it leads to fusion of autophagosomes and lysosomes (Lee et al., 2010). Regarding interplay between autophagy and the UPS, HDAC6 was recently found to be a key molecule in many neurodegenerative diseases. Nevertheless, the functions of HDAC6 in neurodegenerative diseases are controversial. One study showed that deletion of HDAC6 postponed disease progression in a SOD1^G93A^ ALS mouse model (Taes et al., 2013).

In this study, we investigated the potential role of HDAC6 in TDP-43-induced neurotoxicity using mammalian cell models as well as a *Drosophila* model of TDP-43 proteinopathy. We demonstrated that HDAC6 regulates the mislocalization and aggregation of TDP-43. Moreover, we determined that HDAC6 plays a critical role in the deposition of ubiquitinated aggregates and neurotoxicity induced by TDP-43 accumulation through regulation of the ALP pathway in TDP-43 proteinopathy.

## Materials and Methods

### Reagents and Antibodies

The following reagents were purchased from the indicated providers: dimethyl sulfoxide (DMSO; Sigma, D8418) and mifepristone (RU-486; Sigma, M8046). The following antibodies were used for immunoblotting: mouse anti-TurboGFP (Origene, TA150041), rabbit anti-TDP-43 (Proteintech, 10782-2-AP), rabbit anti-LC3 (MBL, PM036), mouse anti-Polyubiquitin (Enzo Life Science, BML-PW8805), mouse anti-Flag (Cell Signaling Technology, 2044), rabbit anti-HDAC6 (Santa Cruz Biotechnology, sc-11420), mouse anti-Lamin A/C (EMD Millipore, 05-714), HRP-conjugated anti-alpha-tubulin (Cell Signaling Technology, 9099), HRP-conjugated anti-rabbit IgG (Santa Cruz Biotechnology, sc-2004), HRP-conjugated mouse IgM (Abcam, ab97230), and HRP-conjugated mouse IgG (Santa Cruz Biotechnology, sc-2005). The following antibodies were used for immunocytochemistry (ICC): rabbit anti-cleaved caspase-3 (CC3) antibody (Cell Signaling Technology, 9664) and Alexa 594-conjugated anti-rabbit IgG (Jackson ImmunoResearch, 111-585-144). The following antibodies were used for immunohistochemistry: rat anti-ELAV (DSHB, RAT-ELAV-7), mouse anti-Polyubiquitin (Enzo Life Science, BML-PW8805), Alexa-488 conjugated rat IgG (Jackson ImmunoResearch, 112-545-167), and Alexa-594 conjugated mouse IgM (Jackson ImmunoResearch, 115-587-020).

### Cell Lines

The Neuro-2a (N2a) mouse neuroblastoma cell line was maintained in Dulbecco’s modified Eagle’s medium (DMEM, Gibco, 11995-065) supplemented with 10% heat-inactivated fetal bovine serum (FBS, Gibco, 16000-044) and 50 μg/ml penicillin-streptomycin (Gibco, 15140-122).

### Transfection

Neuro-2a cells in six-well plates (40 × 10^4^ cells/ml) were transfected with 4 μg of *Gfp* (*pCMV6-AC-Gfp*, Origene Technologies, PS100010) or human *TDP-43* (*pCMV6-AC-TDP-43-Gfp*, Origene Technologies, RG210639) vectors using Lipofectamine 3000 reagent (Invitrogen). Two days after transfection, the knockdown of target proteins was confirmed by immunoblot analysis. For siRNA transfection, N2a cells in six-well plates (40 × 10^4^ cells/ml) were transfected with a control siRNA (Dharmacon; D-001810-10) or mouse *Hdac6* siRNA (Dharmacon; L-043456-02) using Lipofectamine RNAiMAX reagent (Invitrogen). Two days after transfection, the knockdown of target proteins was confirmed by RT-PCR.

### Stable Transfection

Neuro-2a cells in six-well plates (40 × 10^4^ cells/ml) were transfected with 4 μg of human *HDAC6* cDNA using Lipofectamine 3000 reagent (Invitrogen). An empty *pCMV6-Flag* vector was used as a negative control. Stable transfectants were selected in the presence of 800 μg/ml G418 (Gibco, 10131-027). The expression of transgenes was confirmed by immunoblot and ICC analysis.

### Immunoblot Analysis

Cells were homogenized in Cell Lysis Buffer (Cell Signaling Technology, 9803) containing protease and phosphatase inhibitor cocktails. Protein concentrations of the cell lysates were determined by BCA protein assay (Thermo Fisher Scientific, 23225). Next, the protein extracts were mixed with 4× Bolt LDS sample buffer (Invitrogen) and 10× Bolt Sample Reducing Agent buffer (Invitrogen), and then they were boiled at 95°C for 5 min. An equal amount of protein from each sample was separated on Bolt 4–12% Bis-Tris gels (Invitrogen, NW04120BOX) or NuPAGE 3-8% Tris-Acetate gels (Invitrogen, EA0378BOX), and then it was transferred to polyvinylidene difluoride (PVDF, Invitrogen, LC2005) membranes. After blocking membranes with 5% skim milk in TBS with 0.025% Tween 20, blots were probed with antibodies as indicated and detected with an ECL prime kit (GE healthcare, RPN2232). Samples from three independent experiments were used, and the relative expression levels were determined using a Fusion-FX imaging system (Viber Lourmat).

### Preparation of Soluble and Insoluble Cell Extracts

Cells were homogenized in RIPA buffer with protease and phosphatase inhibitor cocktails (Roche, 11836153001, 04906837001). Fractions that were soluble and insoluble in 1% Triton X-100 were obtained by centrifugation at 100,000 × *g* for 30 min at 4°C. Supernatants containing the soluble fractions were harvested, and the pellets for insoluble fractions were solubilized in 2% SDS detergent Cell Lysis Buffer (Cell Signaling Technology, 9803). After sonication, the cell lysates were mixed with 4× Bolt LDS Sample buffer (Invitrogen, B0007) and 10× Bolt Sample Reducing Agent buffer (Invitrogen, B0009), and then they were boiled at 95°C for 5 min.

### Nuclear and Cytoplasmic Extraction

Cells were fractionated using NE-PER nuclear and cytosolic extraction reagents (Thermo Fisher Scientific, 78833). Nuclear and cytoplasmic fractions were obtained in ice-cold CER I and CER II buffer, respectively, by centrifugation at 16,000 × *g* for 5 min at 4°C. Supernatants containing the cytoplasmic extract were harvested, and the pellets were solubilized in ice-cold NER buffer. After vortexing, the extracts were centrifuged at 16,000 × *g* for 10 min at 4°C. Supernatants containing the nuclear extract were harvested. The extracts were mixed with 4× Bolt LDS sample buffer and 10× Bolt Sample Reducing Agent buffer, and then they were boiled at 95°C for 5 min.

### Quantitative RT-PCR

RNA was extracted from cells by using a TRIzol plus RNA Purification Kit (Invitrogen, 12183-555) according to the manufacturer’s instructions. cDNA synthesis was performed at 37°C for 120 min from 100 ng of RNA using a High Capacity cDNA Reverse Transcription kit (Applied Biosystems, 4368814). Quantitative RT-PCR was performed using a one-step SYBR^®^ PrimeScript^TM^ RT-PCR kit (Takara Bio Inc., RR420A) according to the manufacturer’s instructions, which was followed by detection using an Applied Biosystems 7500 Real-Time PCR system (Applied Biosystems). *18S rRNA* and *Gapdh* were used as internal controls. The 2^–ΔΔCt^ method was used to calculate relative changes in gene expression, which were determined by real-time PCR experiments (Livak and Schmittgen, 2001).

### Immunostaining

For ICC, cells were fixed in 4% paraformaldehyde (PFA) in PBS (Gibco, 70011-044) for 30 min at room temperature. The cells were then washed three times with PBS and permeabilized in PBS-T (0.3% Triton X-100) for 15 min at room temperature. After blocking with 5% BSA in PBS-T for 1 h, a rabbit anti-CC3 antibody (1:500 dilution) in 1% BSA in PBS-T was incubated with the cells overnight at 4°C. The cells were then washed three times with PBS and incubated with a secondary antibody [Alexa-594-conjugated rabbit IgG (Jackson ImmunoResearch, 111-585-144, 1:500 dilution)] for 1 h at room temperature. Then, samples were mounted and observed with a fluorescence microscope (Nikon). Photomicrographs from three randomly chosen fields were captured, and the number of CC3^+^ cells was counted among total GFP^+^ cells. For immunohistochemistry, adult flies were dissected in PBS and fixed in 4% PFA in PBS for 30 min at room temperature. The brains were then washed six times with PBS and preincubated in PBS-T for 15 min at room temperature. After blocking with 5% normal goat serum in PBS-T overnight at 4°C, primary antibodies [rat anti-ELAV (DSHB, RAT-ELAV-7, 1:100 dilution) and mouse anti-Polyubiquitin (Enzo Life Science, BML-PW8805, 1:500 dilution)] in 5% normal goat serum in PBS-T were incubated with the tissues for 2 days at 4°C. The brains were then washed six times with PBS and incubated with an Alexa-conjugated secondary antibody for 2 days at 4°C. Alexa 488-conjugated rat IgG (Jackson ImmunoResearch, 112-545-167, 1:500 dilution) and Alexa 594-conjugated mouse IgM (Jackson ImmunoResearch, 115-587-020, 1:500 dilution) were used as secondary antibodies as indicated. The samples were mounted and observed with a fluorescence confocal microscope (Leica). Photomicrographs from three randomly chosen fields were captured, and the number of polyubiquitin^+^ cells was counted.

### Mitochondrial Activity Assay

To assess neuronal mitochondrial dysfunction, *HDAC6-Flag* stable cells were cotransfected with *Gfp* or *TDP-43-Gfp* expression constructs. Two days after transfection, *Gfp*-transfected live cells were subjected to FACS. The sorted *Gfp*-transfected cells (8 × 10^4^ cells/ml) were seeded into XF24-well culture plates (Seahorse Bioscience) and then were allowed to acclimate for 1 day in fresh DMEM. Cells were then washed twice with XF Base Medium supplemented with 2 mM L-glutamine, 10 mM D-glucose, and 1 mM sodium pyruvate (pH 7.4) before being incubated at 37°C in a non-CO_2_ incubator for 1 h. Mitochondrial dysfunction was evaluated using a XF Cell Mito Stress Test Kit (Seahorse Bioscience) according to the manufacturer’s instructions, which was followed by measurement using an XF24 Extracellular Flux Analyzer (Seahorse Bioscience). First, the 24-well utility plate was hydrated, treated with 2 μM oligomycin, 2 μM carbonyl cyanide 4-(trifluoromethoxy) phenylhydrazone (FCCP), 0.5 μM antimycin A/rotenone, and then calibrated by the analyzer. The basal oxygen consumption rate (OCR), ATP production, maximum reserve, and respiratory capacity were calculated as previously described (Dranka et al., 2011), with averages calculated from five wells per condition in each individual experiment. The OCR was normalized to the total protein concentration (OD). After the seahorse analysis, the plate was centrifuged at 280 × *g* for 5 min. The media were aspirated, and the cells were washed twice with PBS. Then, cells were lyzed in RIPA buffer. Protein concentrations of cell lysates were determined using a BCA assay kit.

### Fly Strains

Drosophila stocks were maintained on standard cornmeal agar media at 24°C unless otherwise noted. UAS-TDP-43 and UAS-ATXN2-32Q were described previously (Kim et al., 2014). UAS-HDAC6 has been described previously (Pandey et al., 2007). All other stocks were from The Bloomington Stock Center.

### Climbing and Lifespan Assays

Adult males (0–1 day old) were separated and transferred into experimental vials containing fly media or paper mixed with or without RU-486 (in ethanol, 40 μg/ml) at a density of 25 flies per vial (*n* > 100). The number of dead flies was scored daily, and flies were transferred to fresh media or paper every other day. Adult locomotor function was assessed by a previously described method (Feany and Bender, 2000; Lee et al., 2019), and there were 125 flies per genotype for each time point in all experiments.

### Statistical Analyses

Data were analyzed by Student’s *t*-test (Vassar Stats^1^), one-way ANOVA, or two-way ANOVA test depending on comparison variables, and *post hoc* analysis was performed as indicated (GraphPad Prism Software). Differences were considered significant when *p* < 0.05 and are indicated as follows: *p < 0.05; **p < 0.005; ***p < 0.001; and *n.s.*, not significant.

## Results

### HDAC6 Regulates the Aggregation and Mislocalization of TDP-43

**FIGURE 1:**
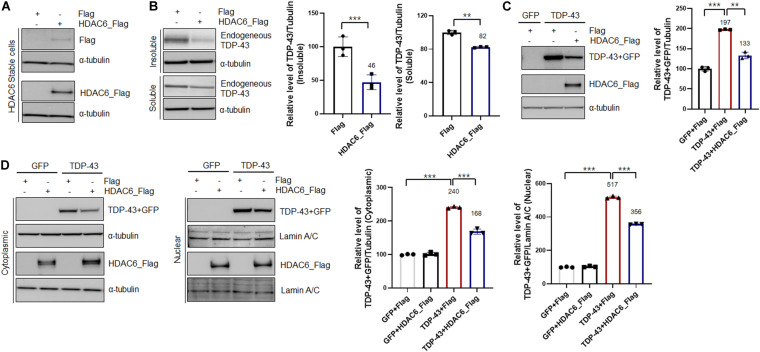
Overexpression of HDAC6 suppresses the aggregation and mislocalization of TDP-43. **(A,B)** Stable Flag- or HDAC6-Flag-expressing cell lines were analyzed by immunoblotting. **(A)** Cell lysates were prepared, and samples were immunoblotted with an anti-Flag or HDAC6 antibody. **(B)** Protein levels of soluble and insoluble TDP-43 in HDAC6-Flag-expressing cells. Flag- or HDAC6-Flag-expressing cells were fractionated to generate a supernatant (the soluble fraction) and a pellet (the insoluble fraction) using lysis buffer containing 1% Triton X-100. Both soluble and insoluble TDP-43 protein levels were significantly decreased by HDAC6-Flag expression. Data are presented as the mean ± SD of three independent experiments. **p < 0.005 and ***p < 0.001 (Student’s *t*-test). **(C,D)** Stable Flag- or HDAC6-Flag-expressing cells were transiently transfected with a plasmid containing either *Gfp* or *TDP-43-Gfp* for 2 days. **(C)** Immunoblot analysis of an anti-TDP-43 or HDAC6 antibody. HDAC6 overexpression markedly decreased the level of TDP-43. Data are presented as the mean ± SD of three independent experiments. **p < 0.005 and ***p < 0.001 (one-way ANOVA with Tukey’s multiple comparison test). **(D)** Protein levels of nuclear and cytoplasmic TDP-43 or HDAC6. HDAC6 overexpression significantly reduced the protein levels of cytoplasmic and nuclear TDP-43 in TDP-43-GFP-expressing cells. Bar graph of the expression levels of cytoplasmic and nuclear TDP-43 normalized to that of tubulin or Lamin A/C. Data are presented as the mean ± SD of three independent experiments. ***p < 0.001 (one-way ANOVA with Tukey’s multiple comparison test).

To investigate the interaction between TDP-43 and HDAC6, we generated a stable N2a cell line expressing *Flag*-tagged *HDCA6*. The expression of HDAC6 was examined by western blotting (Figure 1A). Furthermore, to determine the effects of HDAC6 on TDP-43 aggregation, we separated cell extracts into insoluble and soluble fractions. Strikingly, HDAC6-overexpressing cells showed significantly decreased TDP-43 levels in both insoluble and soluble fractions (Figure 1B). Next, we transfected control *Gfp* or *Gfp*-tagged *TDP-43* into N2a cells that stably expressed either Flag or HDAC6. We found that HDAC6 overexpression clearly reduced total TDP-43-GFP protein levels compared to those of the control (Figure 1C). Moreover, we also observed that HDAC6 overexpression also significantly decreases the protein level of disease-associated TDP-43 mutant (TDP-43^Q331K^-GFP) (Supplementary Figure S1).

The pathological features of TDP-43 proteinopathies are the cytoplasmic mislocalization of TDP-43 and the formation of insoluble TDP-43 aggregates. Indeed, cytoplasmic mislocalization of TDP-43 is a marker of TDP-43-induced toxicity (Kim et al., 2014; Scotter et al., 2015; Shenouda et al., 2018). Thus, to investigate whether HDAC6 regulates the mislocalization of TDP-43, we performed subcellular fractionation to measure the TDP-43 protein levels in both the cytoplasm and nucleus. The protein levels of both cytoplasmic and nuclear TDP-43 were significantly decreased in HDAC6-overexpressing cells (Figure 1D). These results indicate that HDAC6 overexpression mitigates the aggregation and cytoplasmic mislocalization of TDP-43.

**FIGURE 2:**
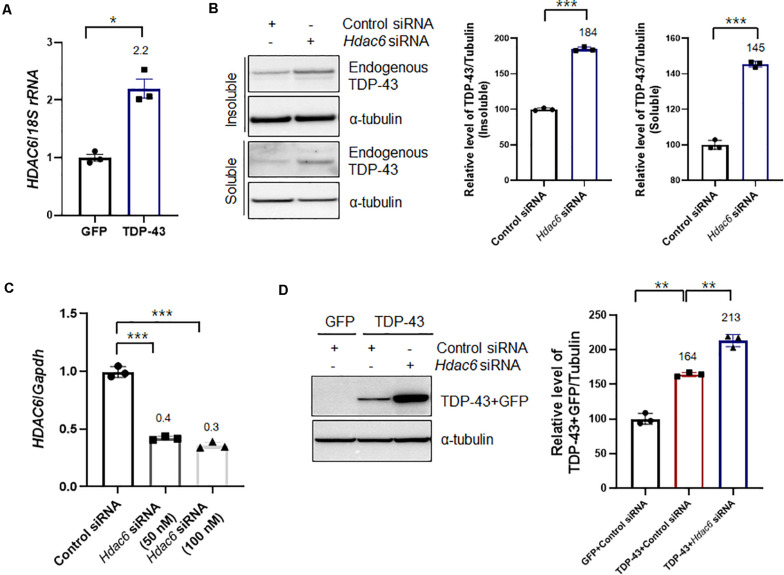
Knockdown of *Hdac6* enhances the TDP-43 pathology in N2a cells. **(A)** N2a cells were transiently transfected with a plasmid containing either *Gfp* or *TDP43-Gfp* and were visualized after 2 days. **(A)** RT-PCR for *HDAC6* mRNA expression in GFP- or TDP-43-GFP-expressing cells. The quantification of *HDAC6* mRNA transcript levels is presented as the mean ± SEM from three independent real-time RT-PCR experiments. *18S rRNA* was used for normalization. *p < 0.05 (Student’s *t*-test). **(B,C)** N2a cells were transfected with control siRNA (100 nM) or *Hdac6*-specific siRNA (50 or 100 nM) for 2 days. **(B)** Protein levels of soluble and insoluble TDP-43 in *Hdac6* knockdown cells. GFP- or TDP-43-GFP-expressing cells were fractionated into the supernatant (the soluble fraction) and the pellet (the insoluble fraction) using lysis buffer containing 1% Triton X-100. Both soluble and insoluble TDP-43 protein levels were significantly increased by TDP-43-GFP expression. Data are presented as the mean ± SD of three independent experiments. ***p < 0.001 (Student’s *t*-test). **(C)** Transfection with an *Hdac6* siRNA efficiently downregulated the mRNA level of *HDAC6* in N2a cells. Data are presented as the mean ± SD of three independent experiments. *Gapdh* was used for normalization. ***p < 0.001 (Student’s *t*-test). **(D)** Control or *Hdac6* knockdown cells were transiently transfected with a plasmid containing either *Gfp* or *TDP-43-Gfp* and were then grown for 2 days. Immunoblot analysis of an anti-TDP-43 antibody. *Hdac6* knockdown markedly increased the level of TDP-43. Data are presented as the mean ± SD of three independent experiments. **p < 0.005 (one-way ANOVA with Tukey’s multiple comparison test).

A previous study showed that TDP-43 directly binds to *HDAC6* mRNA and acts as a regulator of HDAC6 expression (Fiesel et al., 2010; Kim et al., 2010). In line with this evidence, we also observed that TDP-43-overexpressing cells upregulated the expression of *HDAC6* mRNA (Figure 2A) compared to their levels in GFP-expressing cells. Flag-tagged HDAC6 expression did not affect the transcription level of *HDAC6* (Supplementary Figure S2). To further confirm that knockdown of *Hdac6* contributes to TDP-43 protein levels and mislocalization, we downregulated the expression of *Hdac6* by RNAi-mediated gene knockdown. Knockdown of *Hdac6* significantly increased the protein levels of TDP-43 in both insoluble and soluble fractions (Figure 2B). The mRNA level of *HDAC6* was also markedly decreased by *Hdac6* siRNA transfection in N2a cells (Figure 2C). To determine whether HDAC6 modulates TDP-43 pathology induced by TDP-43 overexpression, we measured the levels of TDP-43 protein by immunoblotting after inhibiting *Hdac6* in N2a cells expressing TDP-43. As expected, the levels of TDP-43 protein were significantly increased by *Hdac6* inhibition in TDP-43-overexpressing cells (Figure 2D). We also confirmed these results using a different *Hdac6* siRNA (Supplementary Figure S3). To investigate whether the deacetylase activity of HDAC6 is necessary for HDAC6 mediated TDP-43 regulation, we used tubacin as a specific inhibitor of HDAC6 (Lu et al., 2017). Tubacin treatment did not affect the level of TDP-43 protein in TDP-43-overexpressing neuronal cells. The HDAC6 mRNA level was also not altered in Tubacin-treated cells (Supplementary Figure S4). Taken together, these results suggest that the non-enzymatic function of HDAC6 is implicated in the regulation of TDP-43 pathology.

### HDAC6 Modulates TD-43-Induced UPS Impairment via the Autophagy-Lysosome Pathway

**FIGURE 3:**
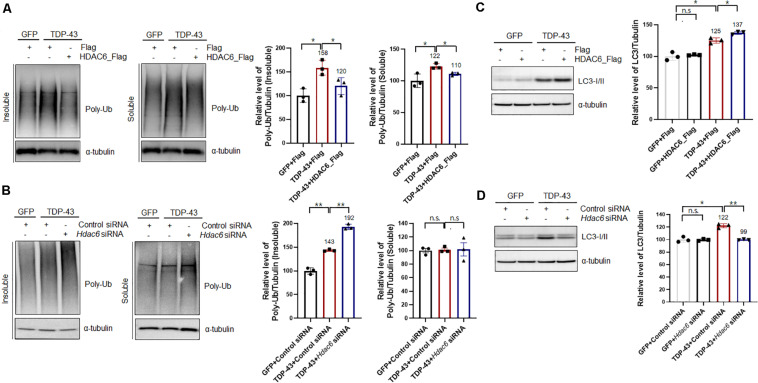
HDAC6 mitigates TDP-43-induced UPS impairment via the autophagy-lysosome pathway. **(A,C)** Stable Flag- or HDAC6-Flag-expressing cells were transiently transfected with a plasmid containing either *Gfp* or *TDP-43-Gfp* and were then grown for 2 days. **(A)** Soluble and insoluble polyubiquitinated protein levels were significantly increased by TDP-43-GFP expression. HDAC6 overexpression significantly reduced TDP-43-induced polyubiquitinated protein levels. Cells were fractionated into the supernatant (the soluble fraction) and the pellet (the insoluble fraction) using lysis buffer containing 1% Triton X-100. Data are presented as the mean ± SD of three independent experiments. *p < 0.05 (one-way ANOVA with Tukey’s multiple comparison test). **(C)** Immunoblot analysis of an anti-LC3 antibody. HDAC6 overexpression markedly increased the level of TDP-43 in TDP-43-GFP-expressing cells. Data are presented as the mean ± SD of three independent experiments. *p < 0.05, *n.s.*, not significant (one-way ANOVA with Tukey’s multiple comparison test). **(B,D)** Control or *Hdac6* knockdown cells were transiently transfected with a plasmid containing either *Gfp* or *TDP-43-Gfp* and were then grown for 2 days. **(B)** Soluble and insoluble polyubiquitinated protein levels were significantly increased by TDP-43-GFP expression. *Hdac6* knockdown significantly increased TDP-43-induced polyubiquitinated protein levels. Cells were fractionated into the supernatant (the soluble fraction) and the pellet (the insoluble fraction) using lysis buffer containing 1% Triton X-100. Data are presented as the mean ± SD of three independent experiments. **p < 0.005, *n.s.*, not significant (one-way ANOVA with Tukey’s multiple comparison test). **(D)** Immunoblot analysis of an anti-LC3 antibody. *Hdac6* knockdown markedly decreased the level of LC3 in TDP-43-GFP-expressing cells. Data are presented as the mean ± SD of three independent experiments. *p < 0.05, **p < 0.005, *n.s.*, not significant (one-way ANOVA with Tukey’s multiple comparison test).

Previous studies demonstrated that HDAC6 modulates the binding affinity of polyubiquitinated proteins, thereby regulating their autophagic degradation (Kawaguchi et al., 2003; Pandey et al., 2007). HDAC6 mediates the sequestration of polyubiquitinated proteins into the autophagosome (Pandey et al., 2007). To determine the effects of HDAC6 on TDP-43-induced UPS impairment, we examined the levels of polyubiquitinated proteins in HDAC6-coexpressing cells and compared them to the levels in cells expressing TDP-43 alone. Consistent with previous findings (Bendotti et al., 2012), TDP-43 overexpression markedly increased the level of polyubiquitinated proteins in insoluble fractions. HDAC6 overexpression effectively decreased the insoluble polyubiquitinated protein levels induced by TDP-43 overexpression, whereas this level was mildly decreased in soluble fractions (Figure 3A). We also confirmed these results using MG132 as an inhibitor for the proteasome. Consistently, MG132 treatment also increased insoluble or soluble polyubiquitinated protein levels, and HDAC6 overexpression significantly suppressed MG132-induced accumulation of ubiquitinated proteins in N2a cells (Supplementary Figure S5). Moreover, HDAC6 overexpression decreased the endogenous TDP-43 protein in soluble and insoluble fraction (Supplementary Figure S6). Furthermore, knockdown of *Hdac6* increased the level of insoluble polyubiquitinated proteins in TDP-43-induced UPS impairment (Figure 3B). Moreover, *Hdac6* inhibition did not affect the level of soluble ubiquitinated proteins following TDP-43-induced UPS impairment (Figure 3B). We also showed that *Hdac6* knockdown clearly increased the endogenous TDP-43 protein in soluble and insoluble fraction (Supplementary Figure S7). Our data suggest that HDAC6 modulates TDP-43-induced UPS impairment in N2a cells.

We next investigated how HDAC6 regulates TDP-43-induced UPS impairment by monitoring the levels of LC3-I/II by western blotting. Interestingly, LC3-I/II levels in TDP-43-overexpressing cells were markedly increased compared to those of control cells. It is already known that the ALP is activated as a compensatory mechanism upon UPS impairment (Wang and Wang, 2015). HDAC6 expression in the TDP-43-expressing cells caused a further increase in LC3-I/II levels (Figure 3C). Moreover, knockdown of *Hdac6* completely abolished the levels of LC3-I/II in TDP-43-expressing cells, as opposed to what was observed in GFP-expressing cells (Figure 3D). We also confirmed TDP-43 protein levels in HDAC6 overexpressing or knock-down cells (Supplementary Figure S8). These results suggest that HDAC6 mediates TDP-43-induced UPS impairment via ALP. To further support this hypothesis, we used Bafilomycin A1 (Baf) as a specific inhibitor of autophagic degradation. We found that HDAC6 overexpression does not affect the level of TDP-43 protein under the condition of ALP inhibition (Supplementary Figure S9). These results indicate that HDAC6 overexpression mitigates TDP-43 induced toxicity via the ALP.

### HDAC6 Attenuates TDP-43-Induced Mitochondrial Dysfunction and Neurotoxicity

**FIGURE 4:**
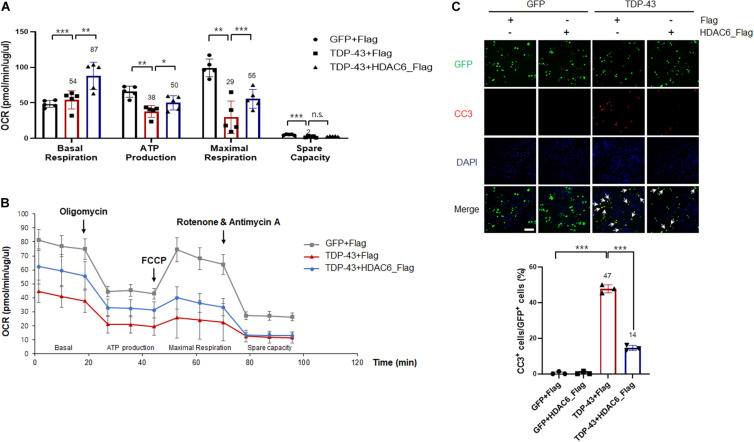
HDAC6 attenuates TDP-43-induced mitochondrial dysfunction and neurotoxicity in N2a cells. **(A,B)** Stable Flag- or HDAC6-Flag-expressing cells were transfected with either *Gfp* or *TDP-43-Gfp* expression constructs, grown for 2 days, and then analyzed. **(A)** Mitochondrial dysfunction analysis of *Gfp*-transfected sorted cells to detect the basal OCR, ATP production, maximum reserve, and respiratory capacity by a Seahorse XF Analyzer. The OCR was normalized to the total protein concentration. **(B)** Quantification of the basal respiration, ATP production, maximum respiration, and spare respiratory capacity is shown as a percentage of the basal values. Data are presented as the mean ± SEM. *p < 0.05, **p < 0.005, ***p < 0.001, and *n.s.*, not significant (two-way ANOVA with Tukey’s multiple comparison test). **(C)** Stable Flag- or HDAC6-Flag-expressing cells were transfected with either *Gfp* or *TDP-43-Gfp* vector were stained for cleaved caspase-3 (CC3; red) or DAPI (nuclei; blue). Arrowheads indicate the colocalization of CC3 with TDP-43-GFP-positive cells. Data are presented as the mean ± SD of three independent experiments. ***p < 0.001 (one-way ANOVA with Tukey’s multiple comparison test). Scale bars, 50 μm.

Recent studies have suggested that mitochondrial damage and dysfunction are pathological features of many neurodegenerative diseases, such as ALS, AD, and Parkinson’s disease (PD) (Shi et al., 2010; Gautier et al., 2014; Birnbaum et al., 2018). The TDP-43-induced mitochondrial defects could be a key characteristic of TDP-43 pathology. Therefore, to investigate whether HDAC6 is implicated in mitochondrial dysfunction caused by TDP-43 expression, we monitored the cellular OCR in real time as a measure of mitochondrial respiration and glycolysis using a Seahorse XF24 Extracellular Flux Analyzer. Sequential injections of oligomycin, FCCP, antimycin A, and rotenone measure basal respiration, ATP production, maximal respiration, and spare respiratory capacity. Notably, we found that basal respiration, ATP production, maximal respiration, and spare respiratory capacity parameters were markedly decreased by TDP-43-expressing cells compared to GFP-expressing cells. The reductions in basal respiration, ATP production, and maximal respiration parameters induced by TDP-43 were greatly ameliorated by HDAC6 overexpression, but spare respiratory capacity was not altered (Figures 4A,B). We next investigated whether HDAC6 regulates TDP-43-induced neurotoxicity, and we measured the levels of CC3 by immunostaining. CC3 is a standard marker for apoptotic cell death (Bressenot et al., 2009). As expected, the CC3-positive cells were greatly increased in the N2a cells expressing TDP-43 compared with the positive cells in the controls. Importantly, TDP-43-induced cell death was more strongly suppressed in HDAC6-expressing cells than it was in Flag-expressing cells (Figure 4C). These findings reveal the possibility that HDAC6 attenuates TDP-43-induced neurotoxicity and UPS impairment via ALP.

### Overexpression of HDAC6 Ameliorates UPS Impairment and Behavioral Deficits in the *Drosophila* Model of TDP-43 Proteinopathy

**FIGURE 5:**
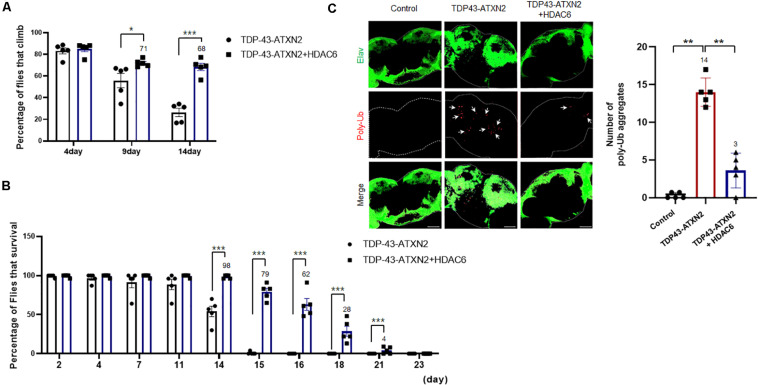
HDAC6 overexpression mitigates neuronal toxicity and ubiquitin aggregates in a fly model of TDP-43 proteinopathies. **(A,B)** Climbing ability and shortened lifespan of TDP-43/ATXN2 or TDP-43/ATXN2+HDAC6 flies at the indicated time-points. The TDP-43/ATXN2-induced motility deficit was significantly rescued by overexpression of HDAC6. Quantification of the percentage of flies that climbed and survived. Data are presented as the mean ± SEM of four independent experiments. *p < 0.05, and ***p < 0.001 (Student’s *t*-test). **(C)** The brains of TDP-43/ATXN2 or TDP-43/ATXN2+HDAC6 flies were immunostained for polyubiquitin (red) and Elav (green). Overexpression of HDAC6 significantly reduced the accumulation of ubiquitinated inclusions in the brains of TDP-43/ATXN2+HDAC6 flies. Elav is a marker of most differentiated neuronal cells in the central and peripheral nervous systems of *Drosophila*. The quantification of the number of polyubiquitin-positive inclusions per brain is shown (*right*). Arrowheads indicate polyubiquitin-positive inclusions. Data are presented as the mean ± SEM of four independent experiments. **p < 0.001 (one-way ANOVA with Bonferroni multiple comparison test). Scale bars, 50 μm. Genotypes: Control is *elavGS/+*, TDP-43/ATXN2 is *UAS-ATXN2-32Q/+; elavGS, UAS-TDP-43/+*, TDP-43/ATXN2+HDAC6 is *UAS-ATXN2-32Q/UAS-HDAC6; elavGS, UAS-TDP-43/+*.

Given the strong *in vitro* evidence that HDAC6 regulates TDP-43-induced UPS impairment and toxicity in N2a cells, we next examined whether overexpressing HDAC6 could suppress TDP-43-induced toxicity *in vivo* using a *Drosophila* model of TDP-43 proteinopathy that expresses human TDP-43 and ATXN2-32Q (Elden et al., 2010; Kim et al., 2014). To test the effect of HDAC6 overexpression on TDP-43/ATXN2 toxicity, we next investigated whether HDAC6 contributes to the restoration of neuronal defects induced by TDP-43/ATXN2 expression in flies. Previously, we showed that flies expressing TDP-43/ATXN2 showed a markedly reduced climbing ability compared to controls (Lee et al., 2019). This climbing deficit and shortened life span were significantly rescued by overexpression of human HDAC6 (Figures 5A,B). These results indicate that TDP-43/ATXN2-induced neuronal toxicity can be suppressed by HDAC6 expression. Furthermore, we also found that HDAC6 expression decreased polyubiquitinated aggregates in brain tissues of TDP-43/ATXN2-expressing flies (Figure 5C). Taken together, we concluded that overexpression of HDAC6 attenuates UPS impairment in TDP-43/ATXN2 flies, which is similar to what was found in the cell-based TDP-43 proteinopathy model. Our data suggest that HDAC6 overexpression ameliorates UPS impairment and behavioral deficits in a *Drosophila* model of TDP-43 proteinopathy.

## Discussion

Understanding HDAC6 function in TDP-43 proteinopathy is crucial for the development of effective treatments for ALS. In this study, we identified HDAC6 as a modulator of cytoplasmic mislocalization and aggregation of TDP-43 in N2a cells. We also found that TDP-43 overexpression increased HDAC6 protein levels. Previously, we revealed that TDP-43-overexpression in neuronal cells dramatically increases the co-localization of polyubiquitinated aggregates and p62 proteins (Lee et al., 2019). Moreover, level of 20S beta5 subunit incorporated into proteasome complex was markedly decreased in TDP-43-expressing cells (Lee et al., 2019). Furthermore, we observed that HDAC6 overexpression mitigates TDP-43-induced UPS impairment via ALP. Importantly, HDAC6 overexpression represses the accumulation of ubiquitinated aggregates in cell models and *Drosophila* models of TDP-43 proteinopathy. Polyubiquitinated proteins can be degraded by ALP under conditions of UPS dysfunction (Korolchuk et al., 2010). Previous studies reported that HDAC6 is a cytoplasmic microtubule-associated deacetylase that mediates the degradation of polyubiquitinated proteins in an ALP-dependent manner (Pandey et al., 2007; Lee et al., 2010). HDAC6 suppresses the toxicity of UPS impairment in *Drosophila* models in an autophagy-dependent manner (Pandey et al., 2007). Moreover, ALP induction enhances the TDP-43 turnover rate and reduces TDP-43-induced neurotoxicity (Wang et al., 2012; Cheng et al., 2015; Leibiger et al., 2018; Nguyen et al., 2018). These results present the possibility that HDAC6 plays an important role in TDP-43 proteinopathy.

Autophagy is a common, major pathway for protein degradation via autophagosome-lysosome fusion in many neurodegenerative diseases. Several studies have shown that HDAC6 plays an important role in autophagosome-lysosome fusion during autophagy. Notably, deletion of HDAC6 resulted in autophagosome maturation failure in an *in vitro* model, resulting in enhanced neurodegeneration (Lee et al., 2010). HDAC6 binds to ubiquitinated proteins via its BUZ domain in an association similar to that of p62/SQSTM1, and the binding occurs under conditions of proteasome impairment (Bjorkoy et al., 2006; Liu et al., 2016). Moreover, HDAC6 interacts with dynein motor protein for transport to aggresomes, a process that is required for autophagic degradation of aggregated proteins (Kawaguchi et al., 2003). To investigate whether HDAC6 regulates autophagic degradation of insoluble ubiquitinated proteins, we examined the levels of polyubiquitinated proteins in insoluble fractions of HDAC6- and TDP-43-coexpressing cells. Intriguingly, overexpression of HDAC6 reduces the accumulation of insoluble polyubiquitinated proteins under conditions of UPS impairment. We also found that HDAC6 overexpression significantly increased the LC3-I/II levels induced by TDP-43 overexpression in N2a cells. Furthermore, we showed that neurospecific expression of HDAC6 decreased the amount of polyubiquitinated aggregates in TDP-43/ATXN2-expressing flies *in vivo*. These results also suggest that HDAC6 facilitates the autophagic degradation of insoluble ubiquitinated proteins in TDP-43 proteinopathy.

Mitochondrial dysfunction is a key pathological feature of ALS pathology. Although TDP-43 accumulation in neurons causes abnormalities in mitochondrial morphology, dynamics, and function in *in vivo* and *in vitro* models (Shan et al., 2010; Xu et al., 2010; Lu et al., 2012; Khalil et al., 2017; Davis et al., 2018), the physiological role of TDP-43 in maintaining mitochondrial function is still unclear. In this study, we observed that HDAC6 attenuates TDP-43-induced mitochondrial dysfunction in N2a cells. TDP-43-induced neurotoxicity is currently a well-known contributor to the pathology of ALS and other neurodegenerative diseases. Cytoplasmic aggregates of TDP-43 have been linked to ALS, FTLD, and AD (Scotter et al., 2015; James et al., 2016; Meriggioli and Kordower, 2016). We showed that TDP-43-induced apoptotic cell death was significantly reduced when TDP-43 was coexpressed with HDAC6. Furthermore, TDP-43-induced neurotoxicity is greatly attenuated by autophagy stimulators in rat cortical neurons (Barmada et al., 2014). Therefore, we concluded that HDAC6 overexpression suppresses TDP-43-induced mitochondrial defects and neurotoxicity via ALP.

## Conclusion

In summary, we demonstrated the following: (1) HDAC6 overexpression decreased the insoluble TDP-43 protein levels in N2a cells; (2) HDAC6 regulates the cytoplasmic mislocalization and aggregation of TDP-43 in N2a cells; (3) HDAC6 mediates TDP-43-induced UPS impairment via ALP; (4) HDAC6 modulates TDP-43-induced mitochondrial dysfunction and neurotoxicity; and (5) HDAC6 overexpression ameliorates UPS impairment and behavioral deficits in a *Drosophila* TDP-43 model. We identified that HDAC6 modulates TDP-43-induced UPS impairment and neurotoxicity via ALP by analyzing TDP-43 overexpression models. Therefore, our results suggest that targeting HDAC6 may represent a novel therapeutic intervention for neurodegenerative diseases with TDP-43 proteinopathy.

## Data Availability Statement

The original contributions presented in the study are included in the article/Supplementary Material. Further inquiries can be directed to the corresponding author/s.

## Author Contributions

SrL, YhK, SyK, MJ, and Y-MJ planned and performed the experiments. SrL, MC, SL, SRK, and KK provided ideas for the project and participated in writing the manuscript. SrL, YhK, KK, and H-JK wrote the manuscript. All authors read and approved the final manuscript.

## Conflict of Interest

The authors declare that the research was conducted in the absence of any commercial or financial relationships that could be construed as a potential conflict of interest.

## Footnotes

1 www.vassarstats.net
