# Supplementary material for: The Role of HDAC6 in TDP-43-Induced Neurotoxicity and UPS Impairment
Source: Front Cell Dev Biol. 2020 Nov 17;8:581942. doi: 10.3389/fcell.2020.581942 (PMC7705063; doi:10.3389/fcell.2020.581942)
Supplement: Supplementary file 1 [file Data_Sheet_1.pdf]

1 Supplementary Methods

1.1 Reagents

The following reagents were purchased from the indicated providers: MG132 (Calbiochem/Merck-Millipore, 474791), Tubacin (Selleckchem, S2239) and Bafilomycin A1 (Baf, Invivogen, tlrl-baf1).

1.2 Transfection

N2a cells in 6-well plates (40×10^4 cells/ml) were transfected with control siRNA (Santa Cruz Biotechnology; Control siRNA, sc-37007), or mouse *Hdac6* siRNA (Santa Cruz Biotechnology; sc-35545) using Lipofectamine RNAiMAX reagent (Invitrogen). After 2 days of transfection, the knockdown of target proteins was confirmed by RT-PCR.

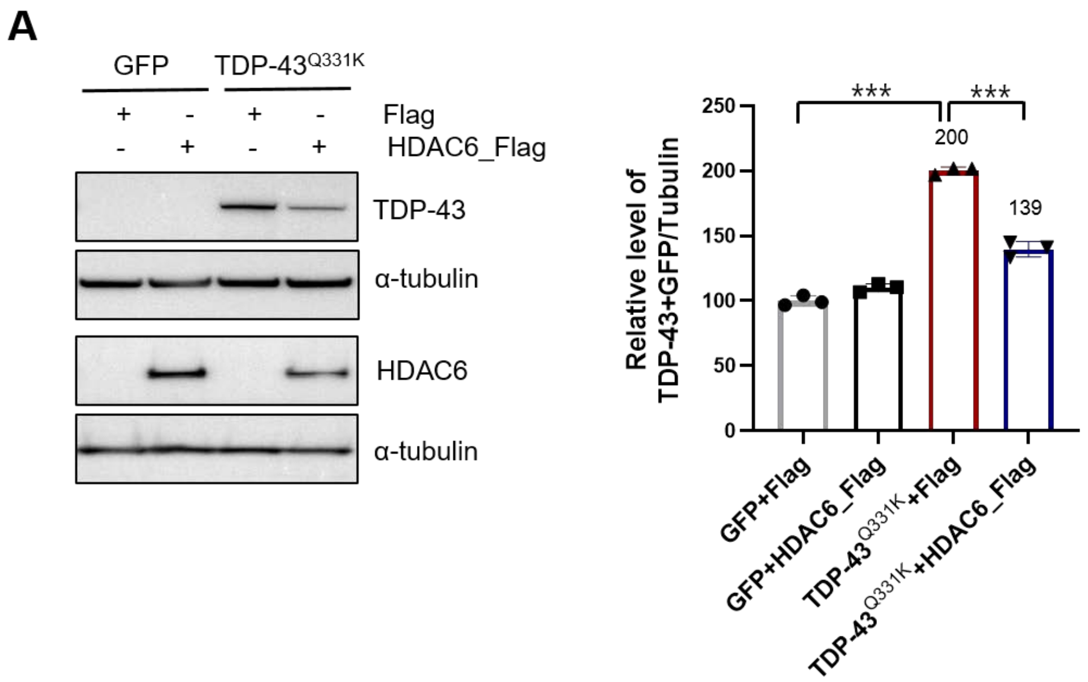

Supplementary Figure S1. HDAC6 overexpression decreases the level of TDP-43^{Q331K} in N2a cells. **(A)** Stable Flag- or HDAC6-Flag-expressing cells were transiently transfected with a plasmid containing either *Gfp* or TDP-43^{Q331K}-*Gfp* for 2 days. Immunoblot analysis of an anti-TDP-43 or HDAC6 antibody. HDAC6 overexpression also significantly decreased the level of TDP-43 in TDP-43 Q331K mutant cells. Data are presented as the mean ± SD of 3 independent experiments. ***p<0.001 (one-way ANOVA with Tukey's multiple comparison test).

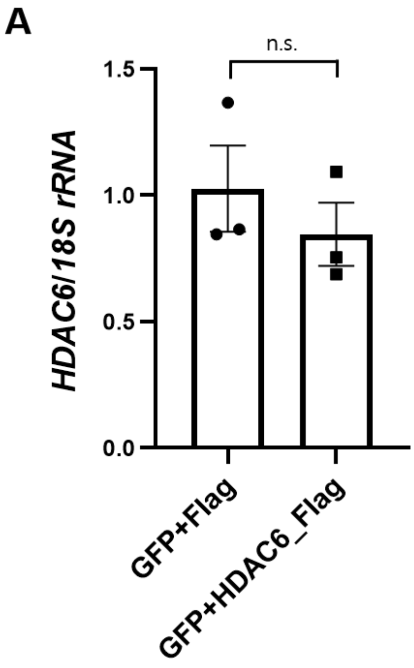

Supplementary Figure S2. HDAC6 overexpression does not affect the transcription level of *HDAC6*. (A) Stable Flag- or HDAC6-Flag-expressing cells were transiently transfected with *Gfp* for 2 days, and then, RT-PCR was performed. The exogenous Flag-tagged HDAC6 expression did not affects level of endogenous Hdac6 protein in GFP-expressing cells. The quantification of *HDAC6* mRNA transcript level is presented as the mean ± SEM from 3 independent real-time RT-PCR experiments. *18S rRNA* was used for normalization. *n.s.*, not significant (Student's *t*-test).

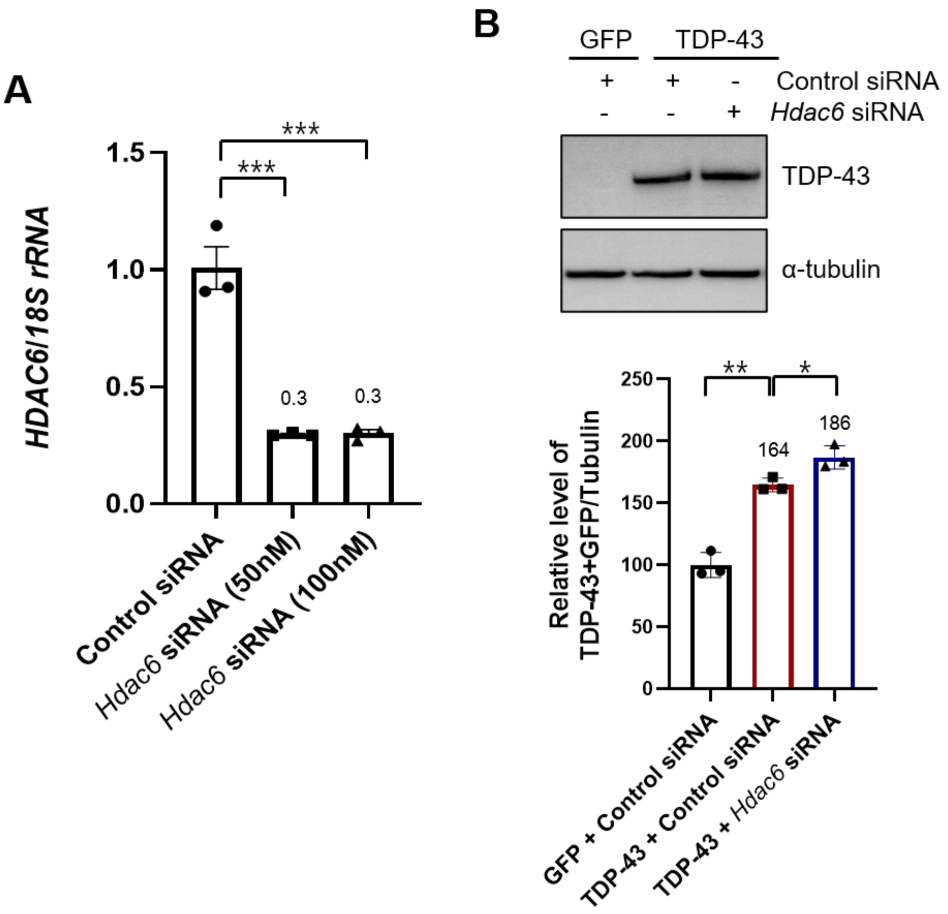

Supplementary Figure S3. Knockdown of *Hdac6* increases the protein level of TDP-43 in N2a cells. (A) N2a cells were transfected with control siRNA (100 nM) or *Hdac6*-specific siRNA (Santa Cruz Biotechnology, 50 or 100 nM) for 2 days. RT-PCR for *HDAC6* mRNA expression in *Hdac6* knockdown cells. The quantification of *HDAC6* mRNA transcript level is presented as the mean ± SEM from 3 independent real-time RT-PCR experiments. *18S rRNA* was used for normalization. ***p<0.001 (one-way ANOVA with Tukey's multiple comparison test). **(B)** Control or *Hdac6* knockdown cells were transiently transfected with a plasmid containing either *Gfp* or *TDP-43-Gfp* for 2 days. Immunoblot analysis of an anti-TDP-43 antibody. *Hdac6* knockdown markedly increased the level of TDP-43. Data are presented as the mean ± SD of 3 independent experiments. *p<0.05, **p<0.005 (one-way ANOVA with Tukey's multiple comparison test).

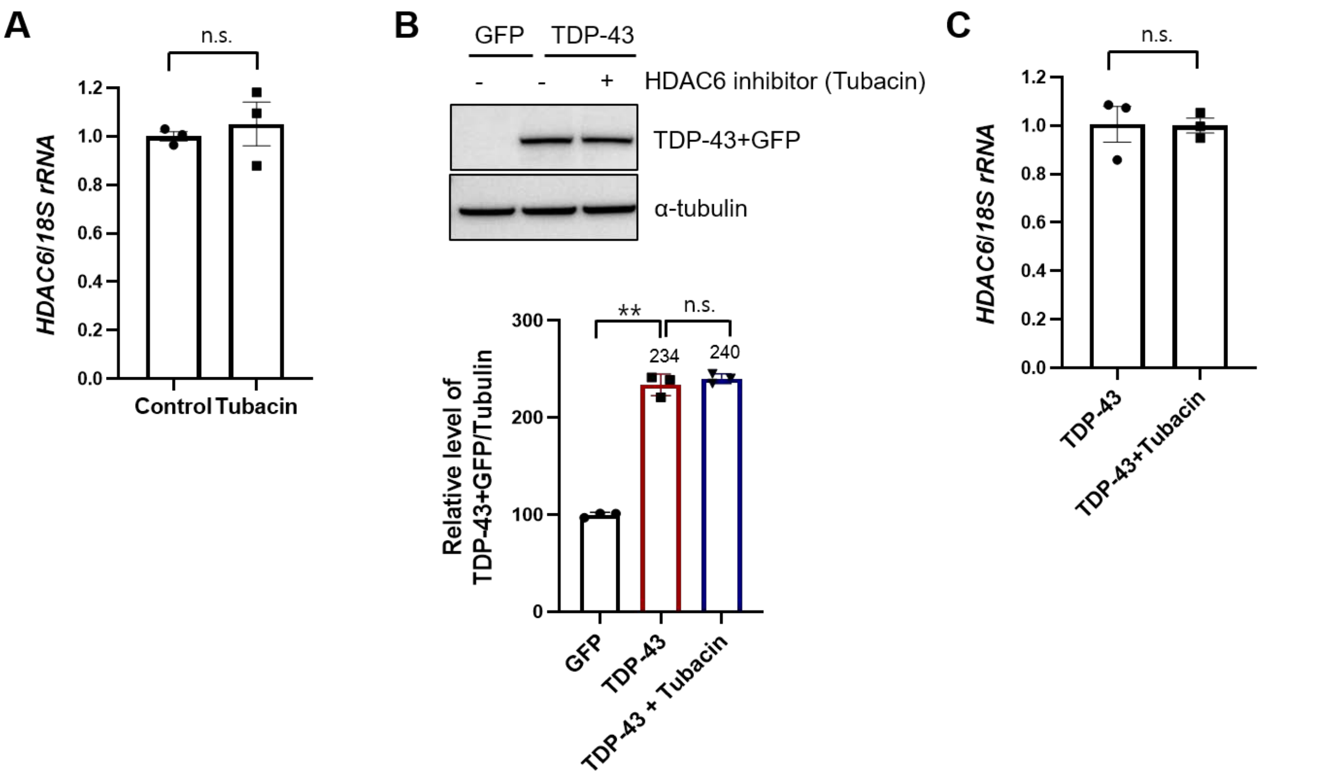

Supplementary Figure S4. Inhibition of HDAC6 deacetylase activity does not affect TDP-43 protein level in N2a cells. (A) N2a cells were treated with HDAC6-specific inhibitors (Tubacine, 1 μM) for 1 day, and then, RT-PCR was performed. The quantification of *HDAC6* mRNA transcript level is presented as the mean ± SEM from 3 independent real-time RT-PCR experiments. *18S rRNA* was used for normalization. *n.s.*, not significant (Student's *t*-test). **(B-C)** N2a cells were pretransfected with *Gfp* or TDP43-*Gfp* for 1 day and subsequently treated with HDAC6 specific inhibitor (Tubacine, 1 μM) for 1 day. **(B)** Immunoblot analysis of an anti-TDP-43 antibody. HDAC6 inhibition does not affect the expression level of TDP-43 in TDP-43-overexpressing cells. Data are presented as the mean ± SD of 3 independent experiments. **p<0.005, *n.s.*, not significant (one-way ANOVA with Tukey's multiple comparison test). **(C)** RT-PCR for *HDAC6* mRNA expression in TDP-43-GFP-expressing cells treated with HDAC6 inhibitor (Tubacine, 1 μM). The quantification of *HDAC6* mRNA transcript level is presented as the mean ± SEM from 3 independent real-time RT-PCR experiments. *18S rRNA* was used for normalization. *n.s.*, not significant (Student's *t*-test).

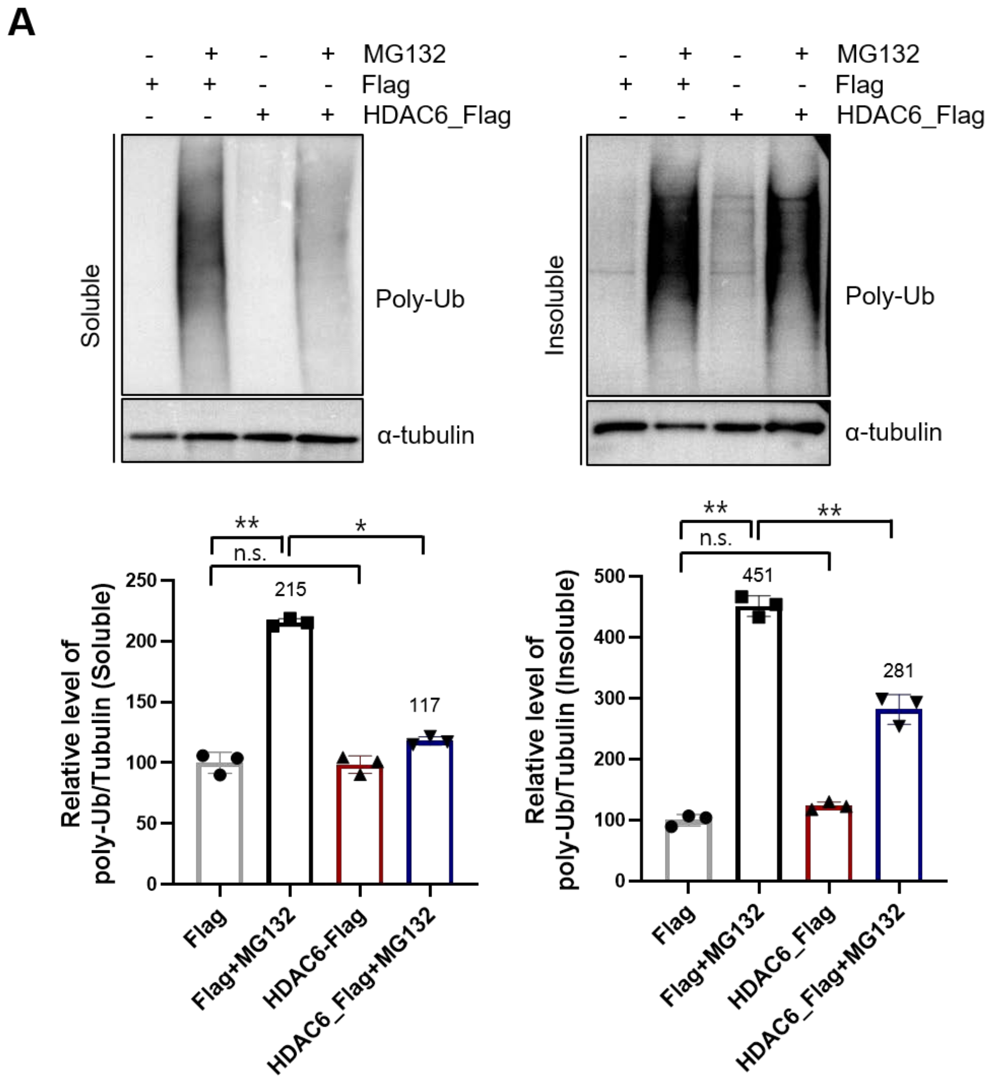

Supplementary Figure S5. HDAC6 overexpression suppresses MG132-induced accumulation of ubiquitinated proteins. (A) Stable Flag- or HDAC6-Flag-expressing cells were treated with MG132 (proteasome inhibitor, 5 μM) for 1 day. Cells were fractionated into the supernatant (the soluble fraction) and the pellet (the insoluble fraction) using lysis buffer containing 1% Triton X-100. Immunoblot analysis of an anti-polyubiquitinated protein antibody. Soluble and insoluble polyubiquitinated protein levels were significantly increased by MG132 treatment. HDAC6 overexpression significantly reduced MG132-induced polyubiquitinated protein levels. Data are presented as the mean ± SD of 3 independent experiments. *p<0.05, **p<0.005, *n.s.*, not significant (one-way ANOVA with Tukey's multiple comparison test).

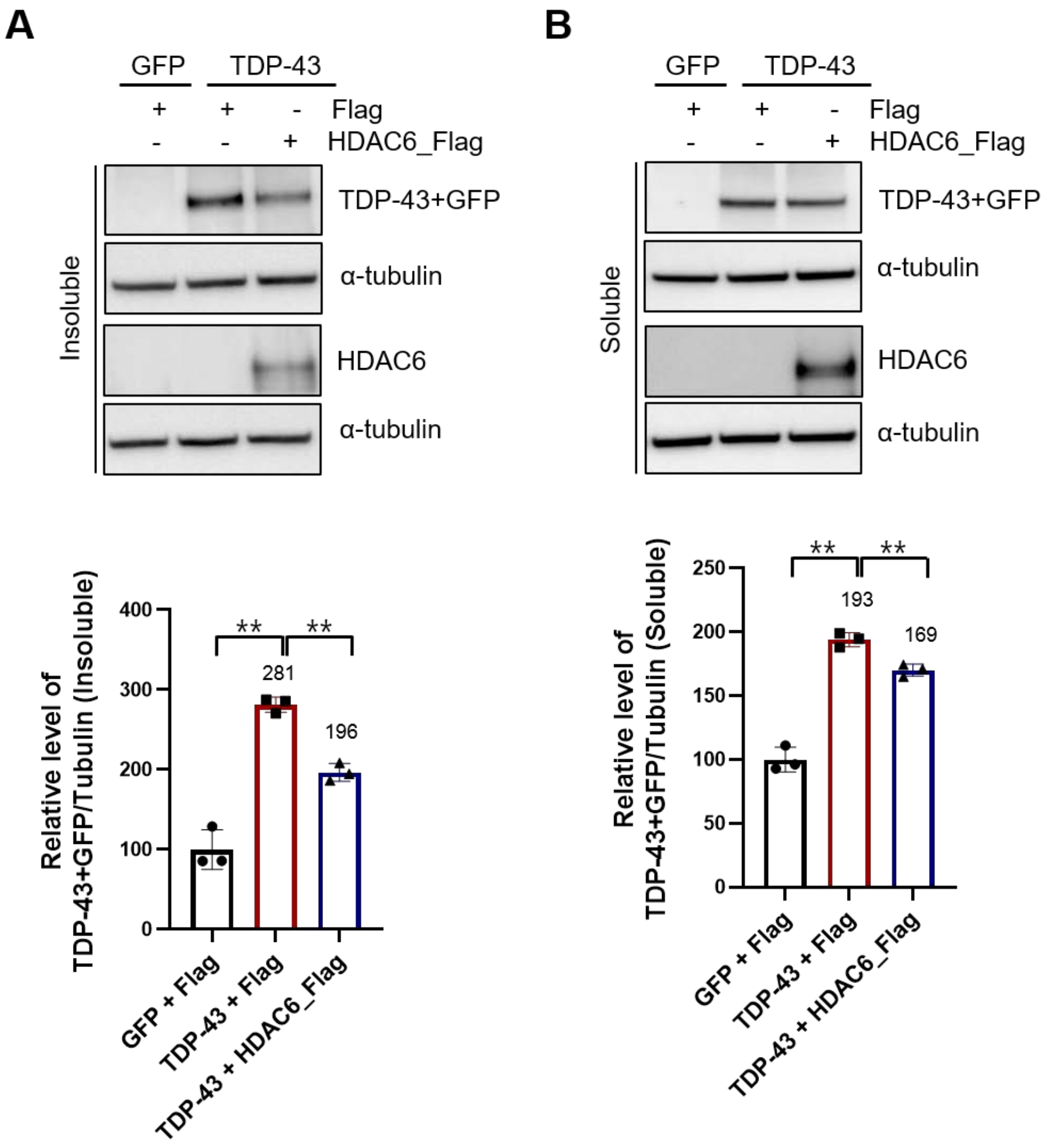

Supple Figure 6. HDAC6 overexpression decreases the levels of insoluble and soluble TDP-43 in N2a cells. (A-B) Stable Flag- or HDAC6-Flag-expressing cells were transiently transfected with a plasmid containing either *Gfp* or TDP-4-*Gfp* for 2 days. Cells were fractionated into the supernatant (the soluble fraction) and the pellet (the insoluble fraction) using lysis buffer containing 1% Triton X-100. Immunoblot analysis of an anti-TDP-43 or HDAC6 antibody. Both insoluble **(A)** and soluble **(B)** TDP-43 protein levels were significantly decreased by HDAC6-overexpressing cells. Data are presented as the mean ± SD of 3 independent experiments. ***p*<0.005 (one-way ANOVA with Tukey's multiple comparison test).

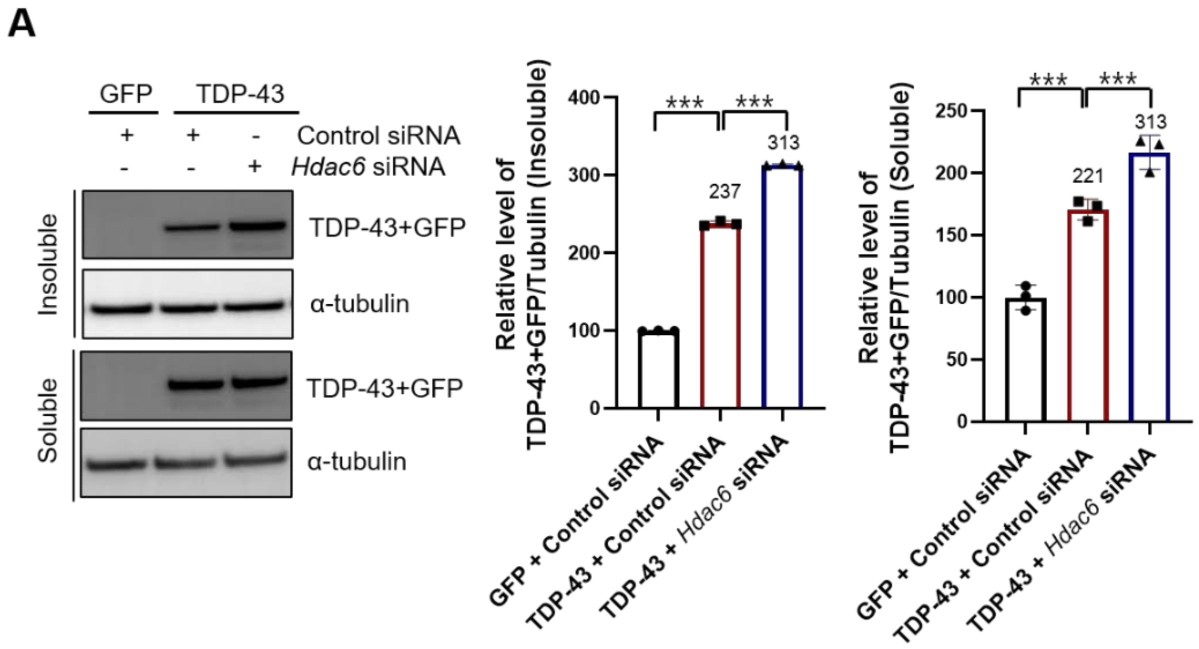

Supple Figure 7. Knockdown of *Hdac6* increases the levels of insoluble or soluble TDP-43 in N2a cells. (A) Control or *Hdac6* knockdown cells were transiently transfected with a plasmid containing either *Gfp* or TDP-43-*Gfp* and were then grown for 2 days. Cells were fractionated into the supernatant (the soluble fraction) and the pellet (the insoluble fraction) using lysis buffer containing 1% Triton X-100. Immunoblot analysis of an anti-TDP-43 antibody. Both insoluble and soluble TDP-43 protein levels were markedly increased by HDAC6-downregulating cells. Data are presented as the mean ± SD of 3 independent experiments. ***p<0.001 (one-way ANOVA with Tukey's multiple comparison test).

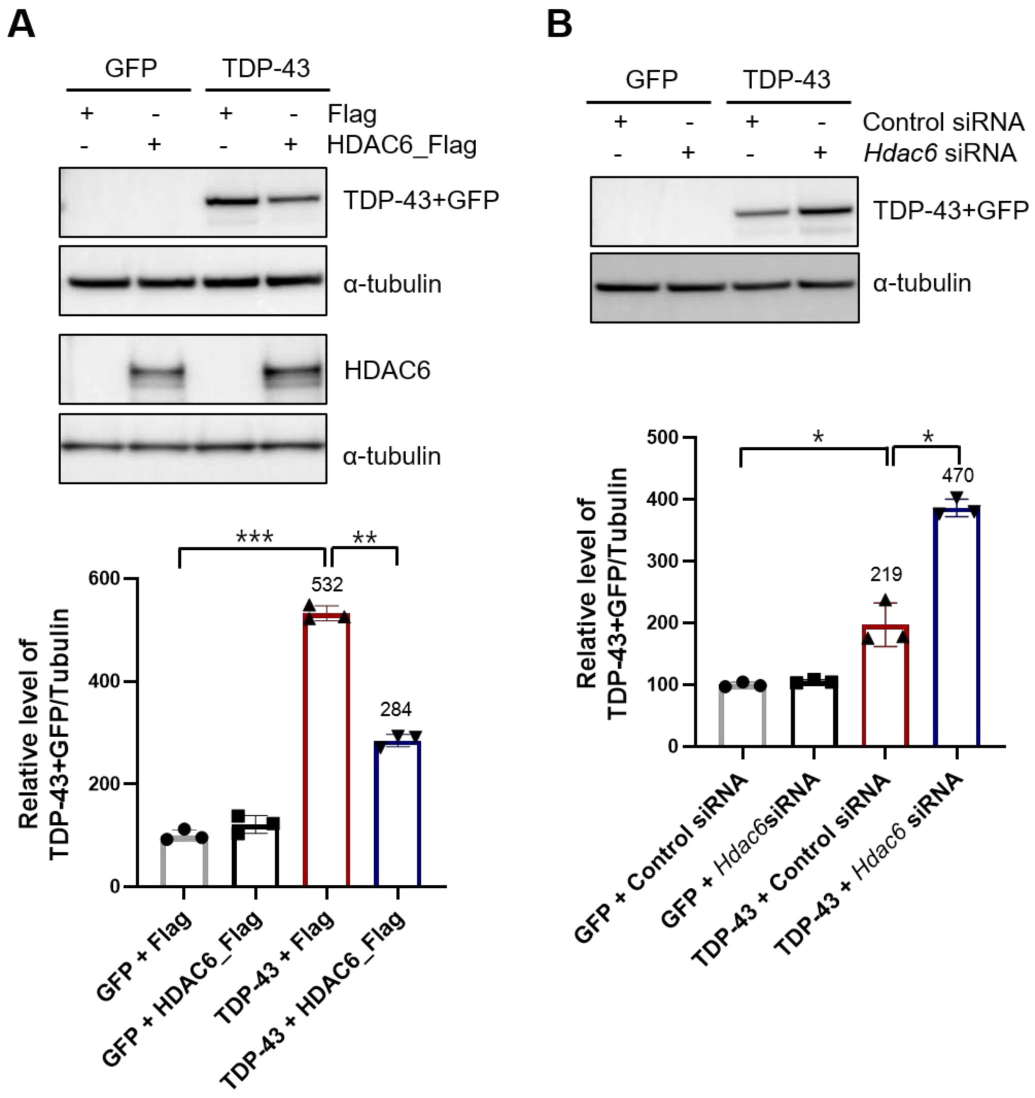

Supple Figure 8. TDP-43 protein level in the HDAC6 up- or downregulated N2a cells. (A) Stable Flag- or HDAC6-Flag-expressing cells were transiently transfected with a plasmid containing either *Gfp* or TDP-4-*Gfp* for 2 days. Immunoblot analysis of an anti-TDP-43 or HDAC6 antibody. Total TDP-43 protein level was greatly decreased by HDAC6-overexpressing cells. Data are presented as the mean ± SD of 3 independent experiments. $^{**}p<0.005$, $^{***}p<0.001$ (one-way ANOVA with Tukey's multiple comparison test). **(B)** Control or *Hdac6* knockdown cells were transiently transfected with a plasmid containing either *Gfp* or TDP-43-*Gfp* and were then grown for 2 days. Immunoblot analysis of an anti-TDP-43 antibody. The TDP-43 protein level was greatly increased by HDAC6-downregulating cells. Data are presented as the mean ± SD of 3 independent experiments. $^{*}p<0.05$ (one-way ANOVA with Tukey's multiple comparison test).

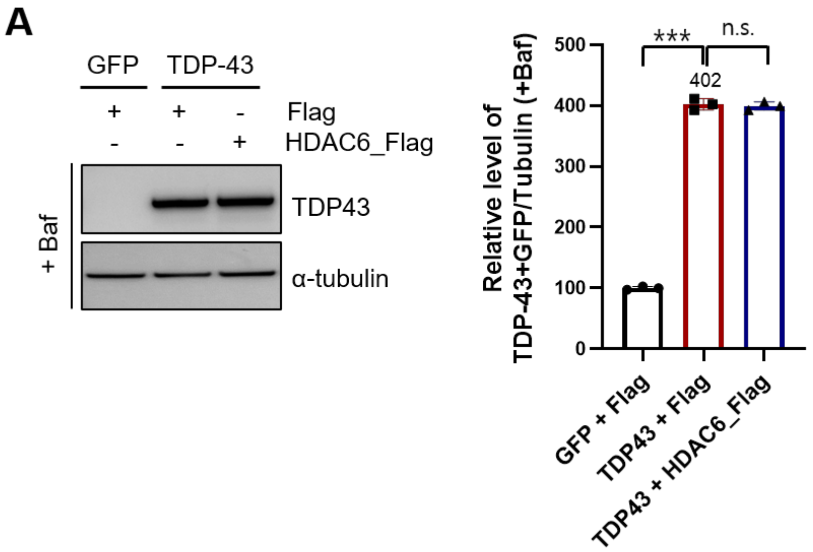

Supple Figure 9. HDAC6 mediated TDP-43 regulation requires ALP. (A) Stable Flag- or HDAC6-Flag-expressing cells were transiently transfected with a plasmid containing either *Gfp* or TDP-4-*Gfp* for 1 day, and then, treated with Bafilomycin A1 (lysosome inhibitor, 2 μM) for 1 day. Immunoblot analysis of an anti-TDP-43 antibody. HDAC6 overexpression did not increase TDP-43 protein level during ALP inhibition. Data are presented as the mean ± SD of 3 independent experiments. ***p<0.001, *n.s.*, not significant (one-way ANOVA with Tukey’s multiple comparison test).
